# Antibacterial Activity of Plant Polyphenols Belonging to the Tannins against *Streptococcus mutans*—Potential against Dental Caries

**DOI:** 10.3390/molecules29040879

**Published:** 2024-02-16

**Authors:** Krzysztof Czerkas, Ewa Olchowik-Grabarek, Magdalena Łomanowska, Nodira Abdulladjanova, Szymon Sękowski

**Affiliations:** 1 Doctoral School of Exact and Natural Sciences, University of Bialystok, 15-254 Bialystok, Poland; 2 Laboratory of Molecular Biophysics, Department of Microbiology and Biotechnology, Faculty of Biology, University of Bialystok, 15-254 Bialystok, Poland; m.lomanowska173@gmail.com (M.Ł.); s.sekowski@uwb.edu.pl (S.S.); 3 Institute of Bioorganic Chemistry, Academy of Sciences of the Republic of Uzbekistan, Tashkent 100143, Uzbekistan; anodira73@rambler.ru

**Keywords:** *Streptococcus mutans*, tannins, dental caries, antibacterial activity, order parameter, fluorescence analysis, zeta potential

## Abstract

Dental caries (DC) is the most common oral pathology. The main bacteria responsible for DC is *Streptococcus mutans*. One of the strategies that can decrease or eliminate the risk of DC development is using compounds that will inhibit both the growth and virulence factors of *S. mutans*. Tannins are plant polyphenols that have strong antibacterial activity. The purpose of this study was to assess the antibacterial activity of three tannins against *S. mutans.* In this investigation, microbiological tests (MIC and MBC) and physicochemical techniques like the fluorescence measurements of tannins’ interaction with *S. mutans* cell membrane and membrane proteins, zeta potential, and thermodynamic analyses were used to obtain knowledge about the antibacterial potential of the investigated compounds against *S. mutans* as well as about the mechanisms associated with antibacterial activity. The obtained results demonstrate that the used compounds exhibit high antibacterial activity against *S. mutans*. The mechanisms of their antibacterial activity are linked to the strong change in the *S. mutans* membrane fluidity and potential, and to their interaction with membrane proteins that can result in great disturbance of bacterial physiology and ultimately the inhibition of bacterial growth, triggering their death. Therefore, it can be concluded that the investigated compounds can be potentially used as natural factors in the prevention of dental caries.

## 1. Introduction

Oral diseases develop mainly due to the activity of different bacterial strains, for example *Lactobacillus fermentum*, *Streptococcus sobrinus*, *Bifidobacterium dentium*, *Treponema denticola*, *Porphyromonas gingivalis*, or *Streptococcus mutans* [1]. 

Among the many oral diseases, one of the most common oral infectious diseases globally is dental caries (DC) [1,2]. DC is a multifactorial oral illness, but the three most crucial factors associated with it are as follows: (i) hygienic, connected with the self-care of teeth and gums, (ii) dietary, and (iii) microbiological, connected with previous bacterial infections [3]. The main pathogen linked with DC is *Streptococcus mutans*. This aerotolerant anaerobes bacterium is characterized by multistep activity, finally resulting in DC development. Its main action is associated with a decrease in pH in the dental oral cavity zone as a result of the production of acid metabolites, leading to demineralization [4], and the synthesis of glucan (by glucosyltransferases enzymes), which enables *S. mutans* adhesion, glucan-binding proteins (GBPs), surface protein antigen c (SPAc), and mutacins (bacteriocins), which are *S. mutans* virulence factors and collagen-binding proteins [5]. Additionally, an important virulence molecule biosynthesized by *S. mutans* is sortase A, a surface protein mainly connected with biofilm formation [6]. It must be emphasized that dental caries and periodontal diseases play a crucial role in oral biofilm formation, leading to demineralization [7] and causing a significant decrease in antibiotic bioavailability to bacteria cells as well as increasing bacteria survival and proliferation [8]. 

Many currently used compounds (e.g., chlorhexidine-CHX) and various study factors (for example, nanoparticles or bacteriophages) against DC act through the inhibition of *S. mutans* biofilm formation [9] and may have documented (like for CHX) or potentially different side effects. According to the well-known adage “prevention better than cure”, the other way to minimize or completely eliminate the risk of DC development is using compounds that will inhibit the growth of *S. mutans* and its virulence factors. This will allow the teeth to be protected against the colonization and biofilm formation of the *S. mutans* bacteria.

Plant polyphenols are very promising compounds that are intensively investigated for their therapeutic potential against different bacteria. These compounds possess antibacterial activity against bacterial strains like *S. aureus*, *P. aeruginosa*, *B. subtilis*, *E. coli*, *K. pneumoniae*, *E. faecalis*, *S. epidermitis*, and others [10]. The wide antimicrobial potential of polyphenols makes them good candidates for therapeutic use against bacteria responsible for oral infectious diseases. It was shown that different polyphenols belonging to flavonoid and tannin groups act against *S. mutans*, *P. gingivalis*, *S. sobrinus*, *S. sanguinus*, and *S. salivarius* [1]. Zayed demonstrated that green tea extracts possess the ability to inhibit *S. mutans* biofilm formation [4]. Another study presented that flavonoids and proanthocyanidins, isolated from cranberry (*Vaccinium macrocarpon*), possess the ability to inhibit the cariogenic virulence factors of pathogens responsible for DC [11]. One polyphenol group of compounds is tannins, i.e., secondary plant metabolites, which despite its high biological activity is less investigated in comparison to other polyphenolic compounds like flavonoids. Tannins possess high antibacterial activity. In our studies, we have demonstrated that PGG (1,2,3,4,6-penta-*O*-galloyl-*β*-d-glucose) as well as 1,2-di-*O*-galloyl-4,6-valoneoyl-β-d-glucose inhibited *S.aureus* growth and can act together with different antibiotics in a synergistic or an additive way [12]. PGG had also the ability to inhibit staphylococcal α-hemolysin release by influencing the bacteria cell and/or *hla* gene expression [13]. The antibacterial activity of tannins is the result of their ability to strongly interact with lipid membranes, leading to changes in their physicochemical properties like fluidity and permeability [12,14,15], and with different proteins like serum albumins [16,17], alpha-synuclein [18], salivary α-amylase [19], and including bacterial ones, e.g., streptococcus glucosyltransferase, protease, or neuraminidase, resulting in their inhibition [20]. Therefore, the aim of these studies was to investigate the antibacterial potential of 1,2,3,4,6-penta-*O*-galloyl-*β*-d-glucose (PGG), 1,2-di-*O*-galloyl-4,6-valoneoyl-*β*-d-glucose (dGVG), and 2-*O*-bis-digalloyl-4,6-valoneoyl-*β*-d-glucose (b-dGVG) against *S. mutans*. Except the standard MIC and MBC values, the antibacterial activity investigations were extended to the measurement of membrane fluidity and integrity, interaction studies with *S. mutans* membrane proteins, and the assessment of the thermodynamic nature of these interactions as well as the analysis of zeta potential at the bacteria surface. Therefore, our work contains the complete study of PGG, dGVG, and b-dGVG against *S. mutans* bacteria.

Our studies possess high novelty. For the first time, we have demonstrated that 1,2,3,4,6-penta-*O*-galloyl-*β*-d-glucose, 1,2-di-*O*-galloyl-4,6-valoneoyl-*β*-d-glucose, and 2-*O*-bis-digalloyl-4,6-valoneoyl-*β*-d-glucose can be utilized as anti-*S. mutans* compounds and potentially used as anticaries agents. 

## 2. Results and Discussion

The investigated compounds, i.e., 1,2,3,4,6-penta-*O*-galloyl-*β*-d-glucose (PGG), 1,2-di-*O*-galloyl-4,6-valoneoyl-*β*-d-glucose (dGVG), and 2-*O*-bis-digalloyl-4,6-valoneoyl-*β*-d-glucose (b-dGVG), possess a similar molecular mass and number of gallic acid residues (5) and glucose moieties (1), as well as hydroxyl groups (15) (Table 1). However, they differ in their molecule’s flexibility due to the absence of a valoneoyl group in the case of PGG and its presence in the case of dGVG and b-dGVG. The valoneoyl group decreases the rotation abilities of gallic acid residues, leading to an increase in the stiffness of molecules. Therefore, the investigations will not only elucidate if the studied compounds possess antibacterial activity against *S. mutans* and connect with it the anticaries potential, but they will also give information about the influence of the valoneoyl group and the molecules’ flexibility on this activity. 

### 2.1. Antibacterial Activity against S. mutans—MIC and MBC Studies

*S. mutans* is a bacterium found in the oral cavity that is strongly linked to the development of dental caries. The bacterium can adhere to and form a biofilm on the surfaces of teeth, acidifying the environment and leading to enamel demineralization [21]. In order to check if the studied tannins—PGG, dGVG, and b-dGVG—have antibacterial potential against *S. mutans*, two parameters were investigated: MIC (minimum inhibitory concentration) and MBC (minimum bactericidal concentration). These two microbiological values are the basic and key microbiological parameters that are most often designated to verify the tested compounds’ antibacterial potential. The obtained results are demonstrated in Table 2.

As observed, the MIC value for PGG was four times lower than for b-dGVG and eight times lower than for dGVG. Therefore, it can be concluded that PGG has the strongest *S. mutans* growth inhibition activity among all tested compounds. When comparing the MBC values, PGG also demonstrated the highest bactericidal activity, which was two times and four times larger than b-dGVG and dGVG, respectively. Also, in our earlier study, PGG showed higher antimicrobial activity against *S. aureus* strain 8325-4 than dGVG [12]. Our results are consistent with data reported in the literature, where isolated polyphenols, including gallic acid, salicylic acid, quercetin, or tannic acid [22] and extracts of polyphenolic compounds [23], showed antibacterial activity against different strains of *S. mutans*. Additionally, the MIC values for the tested tannins against studied *S. mutans* were lower compared to the activity of the *B. crassifolia* extract [24].

The significantly stronger antibacterial activity of PGG compared to dGVG and b-dGVG may result from the compounds having structural differences, leading to varied physicochemical parameters. The same influence of molecular structure on the activity of PGG and dGVG was observed for DMPC liposomes and described in detail in our previous work [25]. Generally, PGG exhibits greater flexibility, has a smaller surface area, higher dipole moment, and greater hydrophobicity than dGVG and, most likely, b-dGVG. Therefore, its enhanced efficacy results from its ability to interact more effectively with the cell membranes of *S. mutans*. Consequently, we continued our investigation into the interactions of tannins with bacterial membranes.

### 2.2. Evaluation of Tannins Interaction with S. mutans Membranes 

**Figure 1:**
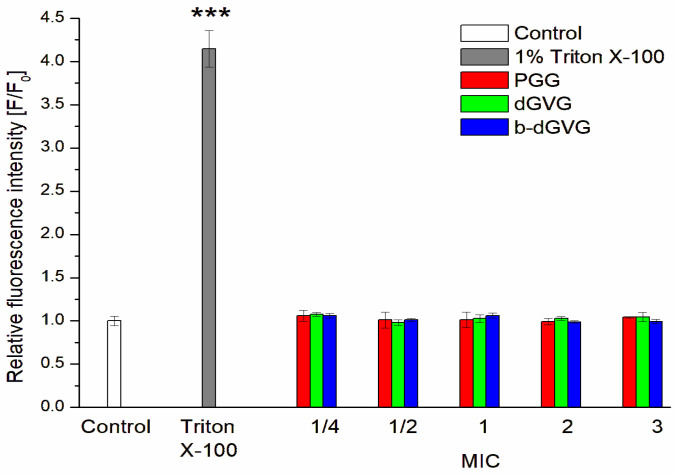
Impact of PGG, dGVG, and b-dGVG on *S. mutans* cell membrane integrity, using 1% Triton X-100 as positive control. The data presented are the means ± SD, n = 6. Statistical significance was estimated using a one-way ANOVA test (results compared to control, *** *p* < 0.001).

One of the potential mechanisms of tannins’ antibacterial activity could be linked to the damage or modification of the cell membrane of *S. mutans*. Bacterial membranes perform critical roles in diverse physiological processes, such as osmoregulation, biosynthesis, transport, and respiration. Consequently, membrane alterations may induce metabolic stress, ultimately resulting in cell death [26]. Many polyphenolic compounds, e.g., kaempferol, quercetin, and apigenin, demonstrate activity against the cell membranes of *S. mutans*, resulting in bacterial death [1]. In the initial phase of evaluating the influence of tannins on the integrity of the *S. mutans* membrane, we used Sytox Green, a marker that binds to DNA when cell membrane damage occurs. The results presented in Figure 1 suggest that bacteria stained with Sytox Green in the presence of PGG, dGVG, and b-dGVG do not exhibit statistically significant changes in membrane integrity compared to the control. In previous studies, *S. mutans* solution without any compounds was used as negative control, whereas *S. mutans* with added Triton X-100, which acts as bacteria membrane-damaging factor [27], was taken as positive control.

To further explore the antibacterial mechanisms of the utilized tannins, we broadened our studies by investigating the compounds’ influence on membrane fluidity. In order to check if analyzed compounds change the membrane rigidity, fluorescence staining was used, and the two fluorescent labels, i.e., TMA-DPH and DPH, which incorporate and anchor to different phospholipid parts of lipid membranes, were applied [25]. TMA-DPH binds to the outer polar layer of the membrane, whereas DPH enters deeper into the hydrophobic region of the phospholipids layer of the membrane. Changes in fluorescence anisotropy were monitored and used to calculate the lipid ordering parameter (S) using Equation (2). 

**Figure 2:**
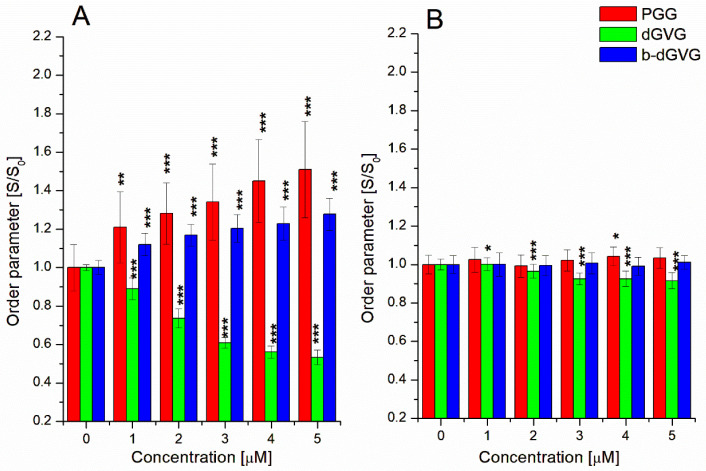
Dependence of the order parameter of *S. mutans* membranes in the presence of tannins measured using TMA-DPH (**A**) and DPH (**B**) probes. The data presented are the means ± SD, n = 6. Statistical significance was estimated using a one-way ANOVA test (results compared to control, * *p* < 0.05; ** *p* < 0.01; *** *p* < 0.001).

Figure 2A presents changes in the ordering parameter at the polar parts of the *S. mutans* membrane in the presence of PGG, dGVG, and b-dGVG. It can be concluded that at concentrations of 1–5 µM, PGG induced significant changes in the ordering parameter for the outer hydrophilic zone of the membrane compared to dGVG and b-dGVG. Interestingly, dGVG resulted in a decrease in the lipid ordering parameter. It was clearly demonstrated that PGG exhibits the strongest interaction with *S. mutans* membranes. Furthermore, the data indicate the stiffening of the polar membrane parts by PGG and b-dGVG, and the increase in fluidity by dGVG.

Subsequently, using Equation (2), the data obtained for DPH-labeled samples were analyzed, and the results are demonstrated in Figure 2B. Changes obtained for PGG were statistically significant for a concentration of 4 µM. In the presence of b-dGVG, very small changes in the *S. mutans* membrane order parameter were statistically insignificant. The opposite effect in comparison with b-dGVG was detected for dGVG, where the 2–5 µM concentration range indicated statistical significance for the increase in fluidity. According to the above results, it can be concluded that dGVG demonstrated the strongest impact on the inner part of the *S. mutans* cell membrane. This suggests that dGVG can penetrate deeper into the lipid bilayer and interact with the hydrophobic part of the *S. mutans* membrane. The strong interaction of tannin molecules with the bacteria cell membrane is attributed to their antibacterial activity. It must be emphasized that this influence manifests in the changes in membrane potential and the modification of membrane fluidity as well as cell membrane disruption [20]. For example, punicalagin modified membrane permeability, whereas proanthocyanidins increased the growth inhibition of bacteria by inactivating ATPase enzymes in the membrane [20]. Also, Delehanty described that proanthocyanidins interacted with the outer bacteria membrane, resulting in the destabilization of their integrity [28].

### 2.3. Evaluation of Tannin Interaction with S. mutans Membrane Proteins through Tryptophan Fluorescence

**Figure 3:**
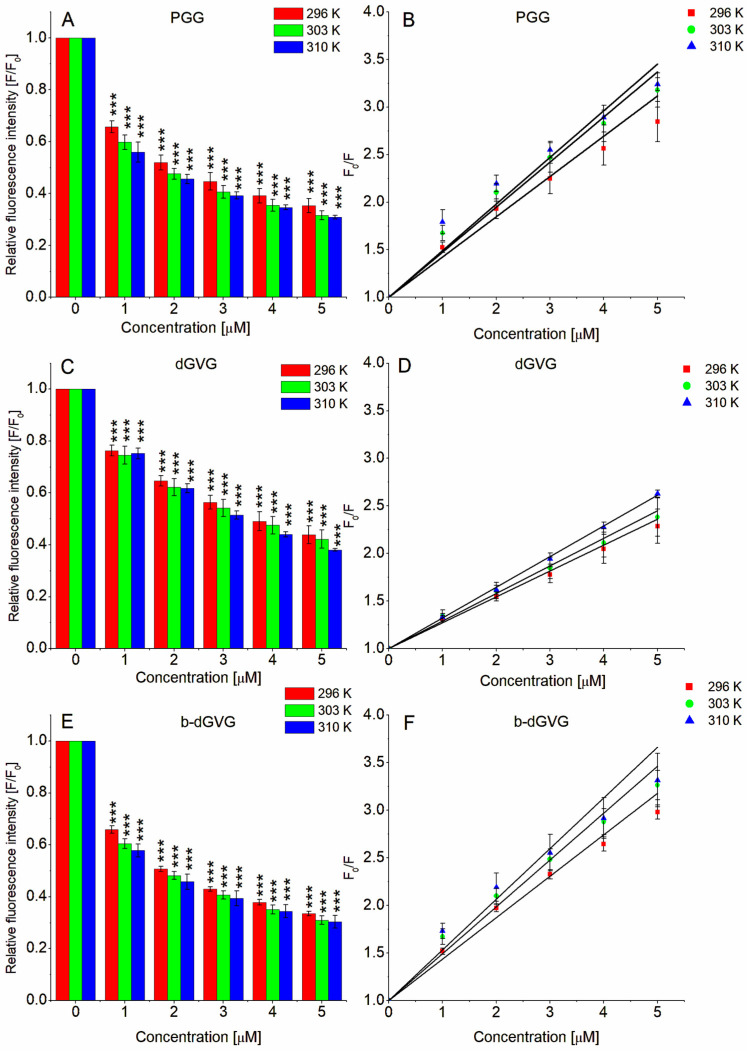
Relative quenching of Trp fluorescence of *S. mutans* membrane proteins in the presence of tannins (**A**,**C**,**E**) and Stern–Volmer plots of Trp fluorescence quenching of *S. mutans* membrane proteins in the presence of tannins (**B**,**D**,**F**). The data presented are the means ± SD, n = 6. Statistical significance was estimated using a one-way ANOVA test (results compared to control, *** *p* < 0.001); K in legend means Kelvin degree.

As we demonstrated above, PGG, dGVG, and b-dGVG can interact with the *S. mutans* cell membrane, modify its fluidity, and influence the membrane order parameter. It is commonly known that the cell membrane has many different proteins that play a crucial role in regulating bacteria’s physiological processes. Tannins have a strong affinity to proteins, forming complexes with membrane proteins and resulting in antibacterial activity [29]. The method enabling the analysis of interactions between different molecules and proteins involves analyzing fluorescence changes descended from aromatic amino acids, including tryptophan. Additionally, tryptophan fluorescence is particularly sensitive to changes in its microenvironment [30]. In order to verify if our analyzed compounds can also interact with *S. mutans* cell membrane proteins, fluorescence investigations were conducted, and the fluorescence signal from the protein tryptophan residues was monitored. Based on these measurements, some physicochemical parameters that characterize these interactions were calculated. The obtained results are presented in Figure 3 and Table 3.

The results allowed us to conclude that tannins decreased the tryptophan fluorescence of *S. mutans* membrane proteins. The effect was concentration- and temperature- dependent (see Figure 3). For the highest concentration at 310 K, a reduction in fluorescence was observed at the levels of 0.309 ± 0.007 for PGG, 0.380 ± 0.005 for dGVG, and 0.303 ± 0.024 for b-dGVG. The results were statistically significance for all tannin concentrations. 

Based on the fluorescence results, the Stern–Volmer plots were drawn (Figure 3B,D,F) and the Stern–Volmer constants (K_SV_) describing the accessibility of the quencher to the quenched molecules (fluorophores) were calculated (see Table 3). As observed, the K_SV_ is larger at higher temperatures, but it should be emphasized that the K_SV_ order of magnitude was not temperature-dependent. As can be observed, PGG and b-dGVG exhibited similar K_SV_ values, while for dGVG, they were approximately 1.5 times lower at each temperature compared to PGG and b-dGVG. This result demonstrates that dGVG had lower accessibility to the quencher (*S. mutans* membrane protein tryptophans). The obtained results correspond very well with the MIC and MBC values and the changes in the physicochemical nature of the *S. mutans* cell membrane. The strongest antibacterial activity (see Table 2) was demonstrated by PGG. This compound’s strongest influence on the *S. mutans* membrane increased the order parameter. The largest PGG activity is the result of the five free galloyl groups. It is well known that the presence of free galloyl moieties is a crucial factor for the antibacterial activity of tannins [20]. Additionally, PGG is highly hydrophobic and thus can penetrate deeper into the membrane in comparison, e.g., with dGVG, as we described earlier [12]. The other compound (b-dGVG), despite being similar to PGG in terms of being accessible to *S. mutans* membrane proteins (b-dGVG K_SV_ values are close to those for PGG), has lower antibacterial activity. This is probably due to the lower number of free galloyl moieties compared to PGG and a lower influence on the membrane order parameter. It should be noted that b-dGVG, in opposition to PGG, possesses a valoneoyl group. This group also determines the antibacterial activity of tannins [20], and as described by Shimozu for two compounds with a valoneoyl group, i.e., isorugosin A and isorugosin B, they showed antibacterial activity against methicillin-resistant *Staphylococcus aureus* (MRSA), and the substitution of two or three galloyl moieties by the valoneoyl group was the factor that induced activity against MRSA [31]. How, then, can we explain that the b-dGVG possesses a more significant activity than dGVG, which has a very similar molecular structure. If it is assumed that the valoneoyl group, which is at the same position attached to glucose of both molecules (i.e., 4,6-valoneoyl-*β*-d-glucose), determines, in the same way, the antibacterial activity, then the explanation should be found in the position of the two remaining galloyl groups. In dGVG, two galloyl moieties are separate and free (1,2-di-*O*-galloyl), whereas in b-dGVG, they are joined together (2-*O*-bis-digalloyl). The configuration of the digalloyl group/groups is also the factor that influences the tannins’ antibacterial activity [20]. Our studies demonstrate that b-dGVG with digalloyl groups has greater anti-*S. mutans* activity in comparison with dGVG, probably resulting from the presence of the digalloyl structure which probably increased antibacterial activity of b-dGVG. It must also be emphasized that this activity is also the result of a stronger interaction with the *S. mutans* membrane and larger modification of the order parameter, as well as the rigidifying of the membrane, contrary to dGVG, which had the weakest interaction with the membrane and evoked an increase in *S. mutans* membrane fluidity. As described above, PGG, dGVG, and b-dGVG possess the ability to interact with *S. mutans* membrane proteins, measured by the tryptophan fluorescence decrease. In order to qualify if tannins decrease Trp fluorescence through complex formation with *S. mutans* proteins (static quenching) or only by collisional encounters (dynamic quenching), the quenching constants (k_q_) were calculated. The obtained k_q_ values for all studied compounds were significantly higher than the maximum scatter collision constant (2 × 10^10^ M^−1^s^−1^), indicating a static quenching mechanism through the formation of tannin–*S. mutans* complexes. Similar results were obtained in our previous studies on PGG and dGVG interaction with membrane proteins of *S. aureus* [12] as well as for staphylococcal alpha-hemolysin [13]. Such strong affinity to *S. mutans* membrane proteins allows us to assume that PGG, dGVG, and b-dGVG inhibited such *S. mutans* proteins, like glucosyltransfrease, surface protein antigen c (PAc) [5], or sortase A (associated with *S. mutans* adhesion, biofilm formation, and evasion of host defense) [6]. 

**Figure 4:**
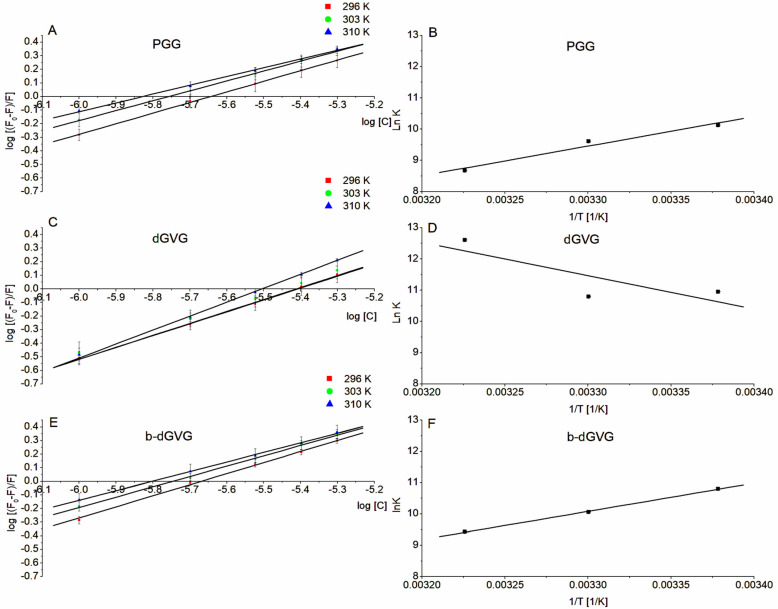
Double logarithmic plots (**A**,**C**,**E**) and Van’t Hoff plots (**B**,**D**,**F**) for tannins–protein interactions of *S. mutans* membranes.

As we demonstrated above, the studied tannins formed complexes with *S. mutans* membrane proteins. To determine whether the formed complexes are reversible or irreversible, the binding constants (logK_b_) were calculated using a double logarithmic plot Equation (5) and drawing double logarithmic plots (Figure 4A,C,E). This enables the estimation of the relative binding strengths between *S. mutans* membrane proteins and tannins at different temperatures. The binding constants for PGG and b-dGVG decreased with temperature, reaching values of 3.769 ± 0.491 for PGG and 4.095 ± 0.116 for b-dGVG at 310 K. In contrast, dGVG exhibited higher values than the PGG and b-dGVG at all used temperatures. Furthermore, an increase in temperature to 310 K resulted in an elevation in logK_b_ to the level of 5.474 ± 0.317. Non-logarithmic values of K_b_ are in the range from 10^4^ to 10^6^ and mostly are in the range 1–15 × 10^4^ M^−1^, indicating the reversibility of such interactions [32]. The results were similar to those obtained for the interaction of four flavonoids with human serum albumin [33].

The interaction between molecules is mainly responsible for hydrogen bonds, van der Waals forces, electrostatic interactions, and hydrophobic forces [34]. Calculating thermodynamic parameters like enthalpy (ΔH) and entropy (ΔS) allows us to qualify which type of bonds (forces) are responsible for molecule–molecule interactions. Hydrophobic bonds predominantly manifest with positive values of ΔH and ΔS, whereas van der Waals forces and hydrogen bonds are associated with negative values of ΔH and ΔS. Electrostatic forces arise when ΔH is negative while ΔS is positive. Additionally, it is possible to assess whether the reaction occurs spontaneously, which is indicated by the negative value of free energy ΔG (Gibb’s potential) [35]. In our studies, the thermodynamic parameters (ΔH and ΔS) were calculated based on fluorescence data and the Van’t Hoff equation (Equation (6)) used to draw Van’t Hoff plots presented in Figure 4B,D,F while the free energy (ΔG) was calculated using Equation (7). Table 4 below demonstrates the values of enthalpy, entropy, and Gibb’s potential. 

According to the obtained results, it can be concluded that the forces responsible for bond formation with bacterial proteins were not the same for all tannins. PGG and b-dGVG interacted with *S. mutans* membrane proteins through van der Waals forces and hydrogen bonds (ΔH and ΔS values were negative). In contrast, for dGVG, both ΔH and ΔS were positive, which clearly suggests that the binding process occurs primarily through hydrophobic interactions. These differences probably result from their different interactions with *S. mutans* membranes. As we observed for PGG and b-dGVG, these two tannins greatly increased the order parameter of membrane polar parts (see Figure 2A) but practically did not influence the order parameter of hydrophobic parts of *S. mutans* membrane (Figure 2B). The opposite effect was detected for dGVG, which decreased the order parameter in both polar and hydrophobic parts of the *S. mutans* membrane (Figure 2), clearly indicating that hydrophobic forces are engaged in such interaction, confirming thermodynamic analyses.

The calculated free energy ΔG was negative for all studied compounds, indicating that PGG, dGVG, and b-dGVG interact with *S. mutans* spontaneously. However, notably, for PGG and b-dGVG, ΔG values decreased with temperature, suggesting a decrease in complex stability. Meanwhile, ΔG values with dGVG increased, indicating an increase in complex stability, which is a known fact as hydrophobic interactions increase protein stability with temperature [36].

### 2.4. Analyses of S. mutans’ Zeta Potential Changes in the Presence of PGG, d-GVG, and b-dGVG 

**Figure 5:**
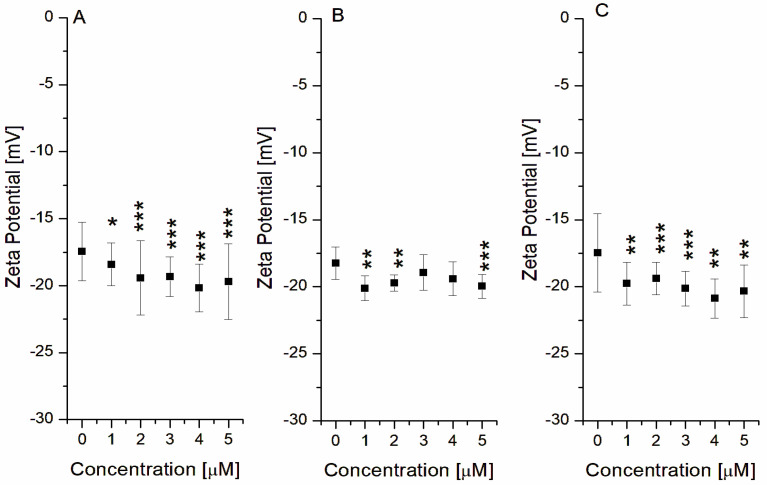
Zeta potential changes in *S. mutans* in the presence of tannins. The data presented are the means ± SD, n = 6. Statistical significance was estimated using a one-way ANOVA test (results compared to control, * *p* < 0.05; ** *p* < 0.01; *** *p* < 0.001). (**A**)—PGG; (**B**)—dGVG; (**C**)—b-dGVG.

One important parameter that is engaged in the interaction between molecules and between molecules and cells is zeta potential (ϛ-potential). Zeta potential is a physicochemical property connected with the cell composition and is one of the crucial factors in the interaction of bacteria with different ions and molecules [37]. Additionally, zeta potential changes in bacterial cells can indicate, among others things, alterations in membrane permeability, biofilm formation, adhesion degree, viability, and the bacteria’s tendency to aggregate under the influence of compounds [38]. Therefore, in our studies, the changes in ϛ-potential have been analyzed as the parameter demonstrating changes at the *S. mutans* charge during interaction with PGG, dGVG, and b-dGVG. The obtained results are presented below (Figure 5).

As it can be observed, the average zeta potential for the control samples of *S. mutans*, i.e., without the addition of tannins, was −17.72 ± 0.454 mV, which, in comparison with the other Gram-positive bacteria such as *S. aureus*, *L. monocytogenes*, and *B. cereus* with zeta potential ranging from −23 to −53 mV, indicates a significantly lower value (if we compare voltage values in mV) [39]. 

The results obtained after the addition of tannins varied slightly. However, they were statistically significant for PGG and b-dGVG for all used concentrations, and the zeta potential negative values gently increased with increasing tannin concentration. Notably, b-dGVG achieved a slightly higher value at concentrations of 4–5 µM. Changes in zeta potential values may be associated with alterations in the order parameter and tryptophan fluorescence quenching of *S. mutans* membrane proteins due to the investigated tannins. The interaction of tannins with proteins and bacterial membranes can lead to changes in zeta potential values, resulting in changes in such parameters as the metabolic and physiological state of bacteria and their capacity for adhesion [38]. For example, compounds like ferulic acid, rosmarinic acid, and epigallocatechin gallate influenced the zeta potential reduction, limiting bacterial adhesion [39].

The above results from our studies demonstrate that PGG, dGVG, and b-dGVG possess antibacterial activity against *S. mutans*. Despite PGG acting the most strongly, dGVG and b-dGVG also showed anti-S. mutans potential. Physicochemical analyses allowed us to detect that the studied tannins change the fluidity of the *S. mutans* membrane and interact spontaneously with bacteria membrane proteins, forming protein–tannin complexes. This observation can have a strong implication for using tannins as natural factors against the development of dental caries. The changes in *S. mutans* membrane fluidity can disturb membrane integrity, and ATPase enzymes can be inhibited as described for the proanthocyanidins [20,25]. Additionally, the complex formation between *S. mutans* membrane proteins and the studied tannins can inhibit essential bacteria proteins like glucosyltransferase, surface protein antigen c, or sortase A, resulting in a decrease in the pathogenicity of *S. mutans*, which will reduce the risk of dental caries.

## 3. Materials and Methods

### 3.1. Chemicals

Studied tannins were isolated from *Rhus typhina* L.—1,2,3,4,6-penta-*O*-galloyl-β-d-glucose (PGG), *Euphorbia jaxartica*—1,2-di-*O*-galloyl-4,6-valoneoyl-β-d-glucose (dGVG), and *P. lanceolata* L.—2-*O*-bis-digalloyl-4,6-valoneoyl-β-d-glucose (b-dGVG). All of the tannins were obtained and isolated by the previously described method [15]. *N*,*N*,*N*-trimethyl-4-(6-phenyl-1,3,5-hexatrien-1-yl) phenylammonium p-toluenesulfonate (TMA-DPH), 1,6-diphenyl-1,3,5-hexatriene (DPH), DMSO, and phosphate buffer saline (PBS) were from SIGMA (Merck); MH broth and MH agar were from Oxoid (Basingstoke, England); Sytox Green was obtained from Thermofisher (Walthman, MA, USA).

### 3.2. Bacterial Strain and Growth Conditions

Studies were conducted using *Streptococcus mutans* ATCC 25175 strain. For investigations, the suspension of bacteria was incubated overnight (t = 37 °C, Mueller–Hinton (MH) broth, 200 rpm continuous shaking). 

### 3.3. Antimicrobial Activity—Determination of Minimum Inhibitory Concentration (MIC) and Minimum Bactericidal Concentration (MBC)

In order to investigate the antibacterial activity of studied tannins against *S. mutans*, two parameters, MIC (minimum inhibitory concentration) and MBC (minimum bactericidal concentration), were analyzed using the broth microdilution method, following guidelines established by the National Committee for Clinical Laboratory Standards. A stock solution of compounds was added to Mueller–Hinton broth (MHB) to achieve a final concentration of 1600 µM. In the next step, the samples were serial two-fold diluted in the MH broth to obtain a concentration range from 800 µM up to 3.25 µM with a final volume of 100 µL per 96-well microtiter plate. Then, 100 µL of *S. mutans* solution with a concentration of 1 × 10^6^ colony-forming units per mL (CFU/mL) was added to each well. The plates were incubated at 37 °C for 24 h to facilitate bacterial growth. The MIC values were identified as the lowest concentration of studied tannins that prevented bacterial growth, indicated by the absence of turbidity. In contrast, MBC was determined as the lowest concentration of used compounds where bacterial growth on the plates had not been observed.

### 3.4. Studies of S. mutans Membrane Permeability—Sytox Staining

Investigations were conducted according to Olchowik-Grabarek et al. [12]. *S. mutans* grew overnight (at 37 °C in Mueller–Hinton broth (MHB) with shaking at 200 rpm). The bacteria solution was centrifuged, and the cell pellet was resuspended in PBS with 5% MH broth, with the final OD_600_ = 0.01. Next, *S. mutans* was pipetted into 96-well plates, and the studied tannins were added to gain the final concentration range of 1/4–3 MIC and incubated for 1 h. The probes with 1% Triton X-100 were prepared as the positive control. Next, the Sytox Green label was added to each well at the final concentration 5 µM. After 2 h of incubation (t = 37 °C), the fluorescence intensity was registered using SpectraMax M2 microplate reader (Molecular Device, San Jose, CA, USA) at the wavelengths λ_exc._ = 504 nm and λ_em._ = 523 nm.

### 3.5. Measurements of S. mutans Membrane Fluidity

Membrane fluidity was measured according to our previous study [12], with some modifications. Briefly, *S. mutans* ATCC 25175 was cultured overnight at 37 °C in Mueller–Hinton broth (MHB) with shaking at 200 rpm. Next, the bacteria suspension was centrifugated (2300× *g* for 10 min), the supernatant was removed, and the bacterial cell pellet was resuspended in PBS buffer (C = 10 mM, pH = 7.4) to achieve *S. mutans* suspension with the optical density OD_600_ = 0.01. The suspension was subsequently labeled (for 15 min, 37 °C) using TMA-DPH or DPH probe at the final concentration of 1 μM, and the fluorescence anisotropy signal was measured using the excitation and emission wavelengths λ_exc_ = 340 nm, λ_em_= 430 nm and λ_exc_ = 348 nm, λ_em_= 426 nm for TMA-DPH and DPH, respectively. Bacteria without studied compounds were taken as control. For analysis, to investigate the tannins’ influence on membrane fluidity, the compounds were added to the labeled *S. mutans* at concentrations of 1–5 μM and incubated for 10 min (t = 37 °C), and the fluorescence anisotropy was read for control. The investigations were conducted using a PerkinElmer LS-55 spectrofluorometer (PerkinElmer, Buckinghamshire, UK). Changes in the fluidity of *S. mutans* membranes induced by tannins were assessed based on the fluorescence anisotropy values of the samples (r). 

The anisotropy values were calculated using the Jablonski equation [15]:

(1) $$r=\frac{I_{VV}-GI_{VH}}{I_{VV}+2GI_{VH}}$$

where I_VV_ and I_VH_ are the vertical and horizontal fluorescence intensities, respectively, for the vertical polarization of the excitation light beam. Before analysis, the G factor (grating correction factor) that corrects the polarizing effects of the monochromator was registered.

The obtained values were used to calculate the order parameter using the equation below [15]:

(2) $$S=\frac{\sqrt{\left[1-2\left(\frac{r}{r_{0}}\right)+5{\left(\frac{r}{r_{0}}\right)}^{2}\right]}-1+\frac{r}{r_{0}}}{2\left(\frac{r}{r_{0}}\right)}$$

where r_0_ is the fluorescence anisotropy of probes in the absence of any rotational motion of probes.

### 3.6. Fluorescence Analysis of Tannins’ Interactions with S. mutans Membrane Proteins

The *S. mutans* cultures were incubated overnight at 37 °C in Mueller–Hinton (MH) broth, with shaking at 200 rpm. For the experiment, bacteria suspension was centrifuged (2300× *g*, 10 min), the supernatant was removed, and bacterial cells were resuspended in PBS (pH = 7.4) to obtain the solution with OD_600_ = 0.1. Next, tannins were added to the bacteria samples at concentrations of 1–5 μM, and the probes were incubated for 10 min at 37 °C. Bacteria without tannins were taken as control. The fluorescence intensities of *S. mutans* membrane proteins were adjusted for appropriate baselines and determined using a PerkinElmer LS-55B spectrofluorometer (PerkinElmer, Waltham, MA, USA). Since the fluorescence descended from the membrane protein tryptophan’s residues, the readings were carried out using the excitation and emission wavelengths λ_exc_ = 295 nm and λ_em_ = 350 nm, respectively. The experiments were conducted at three temperatures (23 °C, 30 °C, and 37 °C).

Fluorescence quenching was characterized using the Stern–Volmer equation [40], and the Stern-Volmer plots were employed for its graphical representation.

(3) $$\frac{F_{0}}{F}=K_{sv}\left[Q\right]+1$$

where: F_0_ and F—fluorescence without and with presence of quencher; K_sv_—Stern–Volmer constant; [Q]—quencher concentration

In order to check which mechanism (static or dynamic) is responsible for both the fluorescence quenching and tannin–protein molecule interactions, the quenching constant (k_q_) [40] was calculated using the following equation:

(4) $$k_{q}=\frac{K_{SV}}{\tau_{0}}$$

where: k_q_—quenching constant; K_SV_—Stern–Volmer constant; *τ*_0_—fluorescence lifetime of fluorophore molecules (5 × 10^−9^ s). 

To determine if the tannin–*S. mutans* interaction is reversible, the binding constant (log K_b_) was calculated based on the double logarithmic plots and double logarithmic equation [41].

(5) $$\log\left[\frac{{(F}_{0}-F)}{F}\right]=\log K_{b}+nlog[Q]$$

where: F_0_ and F—fluorescence without and with presence of quencher; K_b_—binding constant; n—number of binding sites; [Q]—quencher concentration

It is commonly known that all chemical reactions are connected with thermal effects. Analysis of thermodynamic nature of such reaction enables us to determine whether a reaction occurs spontaneously or not [35]. Therefore, to thoroughly explain the nature of PGG, dGVG, and b-dGVG interactions with *S. mutans* membrane proteins, such thermodynamic parameters like enthalpy (ΔH), entropy (ΔS), and free energy change (ΔG) have been calculated using the following equations [35]:

(6) $${lnK}_{b}=-\frac{\Delta H}{RT}+\frac{\Delta S}{R}$$

(7) ΔG=ΔH−TΔS

where: ΔH—enthalpy changes, ΔS—entropy changes, ΔG—free energy changes, T—temperature at Kelvin scale, R—gas constant, and lnK_b_—natural logarithm of binding constant. 

### 3.7. Analysis of ϛ-Potential 

Changes in the zeta potential (ϛ-potential) of *S. mutans* in the presence of the studied compounds were measured using electrophoretic light scattering with a zetasizer ULTRA (Malvern, Worcestershire, UK). Bacteria cell suspension was prepared in the same way as for fluorescence analyses. Next, 3 mL of the *S. mutans* was incubated with tannins in the concentration range of 1–5 μM for 15 min at 25 °C. The analyses were performed at a temperature of 25 °C in disposable folded capillary cells (DTS1070 Malvern Panalytical Ltd., Malvern, UK).

### 3.8. Statistical Analysis

A minimum of three independent trials were conducted for each of the experiments. The data are presented as the mean ± standard deviation. Grubb’s test was applied to ensure that the raw data values did not deviate significantly from the overall dataset. Statistical analysis was conducted using the GraphPad software QuickCalc. Graphs and figures were created using Origin 8.5.1 software (Northampton, MA, USA).

## 4. Conclusions

One of the most serious oral pathologies is dental caries (DC). Among many reasons responsible for DC development, the main one is *S. mutans* infection. This bacterium is responsible for demineralization, glucan synthesis, pH change, and biofilm formation, which results in caries development at the final stage. Tannins, natural plant compounds, have well-documented antibacterial activity. Therefore, our studies investigated the antibacterial potential of three tannins against *S. mutans*. Using microbiological and physicochemical analyses, we demonstrated that studied compounds possess a strong affinity to *S. mutans* bacteria cells and demonstrate quite high antibacterial activity. Among the tested compounds, the highest antistreptococcal activity was observed for PGG. The observed antibacterial potential of PGG, dGVG, and b-dGVG was connected with modifications of *S. mutans* membrane fluidity and membrane surface charge, as well as with strong interactions with bacteria surface proteins and the protein–tannin complex formation. Thermodynamic analysis shows that all tannins bound to bacteria membrane proteins spontaneously. According to the obtained results, it can be concluded that tannins are efficient anti-*S. mutans* compounds and can be potentially used as natural protection against caries, but further research is needed to develop the most efficient systems to apply tannins in the oral cavity space.

## Figures and Tables

**Table 1:** Chemical structures and basic properties of PGG, dGVG, and b-dGVG.

	PGG	dGVG	b-dGVG
Chemical structure	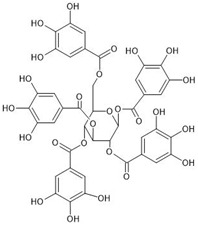	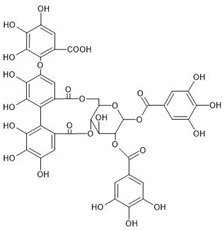	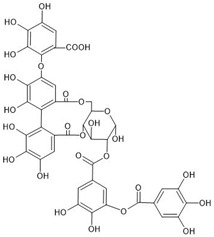
Chemical formula	C_41_H_32_O_26_	C_41_H_30_O_27_	C_41_H_30_O_27_
Molecular weight	940.681 g/mol	954.664 g/mol	954.664 g/mol
Number of hydroxyl groups (-OH)	15	15	15
Number of gallic acid residues	5	5	5
Number of glucose moieties	1	1	1
Origin	*Rhus typhina* L.	*Euphorbia E. turkestanica*	*Plantago lanceolata* L.

**Table 2:** Antibacterial activity (MIC and MBC) of PGG, dGVG, and b-dGVG against *S. mutans*.

Tannin	MIC [µM]	MBC [µM]
PGG	50	200
dGVG	400	800
b-dGVG	200	400

**Table 3:** Binding parameters of tannin—*S. mutans* membrane protein interactions (K_SV_—Stern–Volmer constant, k_q_—quenching constant, and logK_b_—logarithm of binding constant).

		Binding Parameters		
Tannin	Temperature [K]	K_sv_ [×10^6^] [M^−1^]	k_q_ [×10^14^] [M^−1^·s^−1^]	logK_b_
PGG	296	0.393 ± 0.045	0.786 ± 0.090	4.398 ± 0.404
	303	0.464 ± 0.042	0.927 ± 0.085	4.175 ± 0.230
	310	0.484 ± 0.020	0.967 ± 0.040	3.769 ± 0.491
dGVG	296	0.261 ± 0.030	0.522 ± 0.068	4.758 ± 0.216
	303	0.281 ± 0.039	0.562 ± 0.079	4.689 ± 0.186
	310	0.321 ± 0.010	0.642 ± 0.020	5.474 ± 0.317
b-dGVG	296	0.417 ± 0.016	0.834 ± 0.032	4.692 ± 0.240
	303	0.468 ± 0.032	0.937 ± 0.063	4.368 ± 0.416
	310	0.4914 ± 0.059	0.983 ± 0.117	4.095 ± 0.116

**Table 4:** Thermodynamic parameters of the binding of tannins with *S. mutans* membranes.

Tannin	T(K)	ΔH (kJ·mol^−1^)	ΔS (kJ·mol^−1^K^−1^)	ΔG (kJ·mol^−1^)
PGG	296	−78.7697	−0.18131	−25.1026
	303			−23.8335
	310			−22.5643
dGVG	296	89.11174	0.389428	−26.1589
	303			−28.8840
	310			−31.6109
b-dGVG	296	−74.8819	−0.16328	−26.5519
	303			−25.4089
	310			−24.2660

## Data Availability

Data is contained within the article.
